# Empowering Advanced Practice Nurses: A Review of Addressing Global Health Needs

**DOI:** 10.5334/aogh.4723

**Published:** 2025-08-13

**Authors:** Carole Mackavey, Colette Henderson, Gillian Morris

**Affiliations:** 1 University of Texas Health Science Center @Houston Cizik SON, Texas, USA; 2 School of Health Sciences, University of Dundee, Scotland

**Keywords:** global health, advanced practice nursing, global health outcomes

## Abstract

*Aims:* This paper explores the current global health landscape and the transformative potential of empowering Advanced Practice Nurses (APNs) to address global health challenges. It highlights successful models of APN integration from various countries, showcasing their positive impact on patient outcomes and the efficiency of the healthcare system.

*Introduction:* This narrative provides an overview of the current impact of APNs and Nurse Practitioners (NPs) on global health, as well as areas where APNs/NPs can enhance healthcare outcomes. It emphasizes the crucial role that APNs play in addressing healthcare issues and outlines their potential to expand access to quality care through their practice and leadership.

*Methods:* A review of the literature examines current trends and research; this paper highlights critical areas where APNs can make substantial contributions, such as chronic disease management, primary care access, emergency care, and overall health system improvement.

*Results:* It is essential to optimize the utilization of APNs/NPs, standardize APN roles and scope of practice internationally, enhance APN educational programs, and promote interprofessional collaboration.

*Conclusion:* The global healthcare landscape faces unprecedented challenges, including aging populations, increasing burdens from chronic diseases, and persistent health disparities. By empowering APNs, healthcare systems can better address the diverse and evolving health needs of global populations.

*Impact:* APNs and NPs can significantly and multifacetedly impact global health. They are uniquely positioned to provide holistic and patient‑centered care, improve access to services, mitigate provider shortages, enhance quality, and address health disparities.

*Nursing contribution:* APNs/NPs are underutilized in healthcare. They are experts in health promotion and disease prevention, both of which are crucial for improving global population health. APNs/NPs can contribute to achieving the United Nations Sustainable Development Goals (SDGs) related to improved health, greater gender equality, and stronger economies.

## Aim

The primary aim of this review is to explore and explain the essential role of Advanced Practice Nurses (APNs) and Nurse Practitioners (NPs) in addressing global health challenges. Examining current practices, innovative strategies, and educational frameworks highlights how APNs can be empowered to effectively meet the diverse and evolving health needs of populations worldwide.

## Introduction

With justice and human rights as its foundation, global health is an area of study, research, and practice that prioritizes achieving health and health equity for all people worldwide. The field of global health is rapidly evolving, and the demand for skilled healthcare professionals is increasing worldwide. The following definition of global health by J.P. Koplan et al. [1] is widely accepted, although there is no agreed definition, and the concept continues to be debated.

Global health is an area for study, research, and practice that places a priority on improving health and achieving equity in health for all people worldwide. Global health emphasizes transnational health issues, determinants, and solutions; involves many disciplines within and beyond the health sciences and promotes interdisciplinary collaboration; and is a synthesis of population‑based prevention with individual‑level clinical care.

APNs and NPs play a crucial role in addressing the growing healthcare needs of diverse populations worldwide. With their holistic approach, advanced clinical expertise, and commitment to preventive care, APNs and NPs are well‑positioned to contribute significantly to global health initiatives. They actively expand healthcare access, respond to emergencies, improve health outcomes, and address healthcare disparities [2].

APNs have the potential to help offset the shortage and maldistribution of healthcare providers around the world. Their expanded scope of practice, including holistic assessing, diagnosing, ordering tests, prescribing medications, and empowering patients to improve their health and well‑being, allows them to provide comprehensive care to individuals and communities. APNs can serve patients and communities in complex and person‑centered ways when systems of education and healthcare delivery are thoughtfully designed. As the global community works toward universal healthcare coverage, APNs should be a meaningful part of the solution [3].

## Background: The Global Health Landscape

Global health challenges are multifaceted and complex, ranging from infectious diseases and maternal and child health issues to non‑communicable diseases and access to healthcare. However, the COVID‑19 pandemic has not just added to these challenges but has cast a long and dark shadow over them. It has had a significant negative impact on life expectancy worldwide, reversing decades of progress in improving longevity in some places [4]. The effect was uneven across regions, with the Americas and Southeast Asia experiencing the most significant setbacks, with life expectancy dropping by approximately three years between 2019 and 2021 [5]. This represents the first decline in global life expectancy in nearly 30 years. The COVID‑19 pandemic has effectively eliminated almost a decade of progress in improving global life expectancy levels, underscoring the profound impact of the crisis on population health and longevity worldwide. Socioeconomic disparities, political instability, and limited resources in many world regions have exacerbated numerous health issues.

The COVID pandemic was also the catalyst for many APN innovations in healthcare delivery [6]. Given the severity and complexity of these global health challenges, advanced practice providers, particularly APNs, are well‑equipped and essential in fighting them [3, 6]. Their comprehensive, holistic, and collaborative approach to healthcare is a key component and a driving force in this fight.

Non‑communicable diseases (NCDs) currently account for 74% of global mortality, and 77% of those are in low‑to‑middle‑income countries (LMICs) [5]. Unwin [7] identified lifestyle factors as the root cause of many NCDs, which challenge overwhelmed healthcare systems across the world. Global action and collaboration are essential to halt the current disease crisis. APNs are experts in disease prevention, as well as the diagnosis and management of a range of conditions; they are well‑placed to lead interventions that can support improvement in health behaviors and better metabolic health through lifestyle changes. Encouraging people to adopt a healthy diet, increased physical activity, and adequate sleep has the potential to reduce the levels of NCDs significantly [8]. All healthcare professionals require an understanding of the broader influences of social determinants and how they influence health. However, there is no quick fix for these interventions. In pressurized healthcare systems, there may be little time to factor in interventions such as motivational interviewing, brief intervention, or coaching.

The cornerstone to supporting lifestyle changes is skilled and empathetic communication, which rejects any paternalism and embraces empowerment through shared decision‑making [9]. APNs have these skills, but perhaps there needs to be a reframing of the approaches to clinical management of people, which improves health through lifestyle changes, including dietary modifications, increased physical activity, stress management, and sustainable behavioral changes [8]. Adopting an approach with a focus on diagnosis rather than on the individual perpetuates a power imbalance, which leads to patients not engaging in health improvement initiatives [10]. These interventions are key to helping individuals maintain a healthy metabolic state with high insulin sensitivity and low vulnerability to metabolic conditions like obesity and type 2 diabetes mellitus [8]. There is an opportunity for APNs to lead and contribute to significant health improvement across the world by factoring in lifestyle changes as part of their routine care.

Stefan, Birkenfeld, and Schulze [11] advise that impaired metabolic health is an essential determinant of the severity of COVID‑19, increasing complications and mortality, and it may also reduce the efficacy of vaccines. This global health concern remains under‑researched, and its definition is contentious due to the lack of focused research in this area [12]. Described as a global and “major health hazard of the modern world” [13], health promotion activities would diminish the economic and health costs of this malady. In addition, researchers at Oxford Population Health found an association between poor metabolic health and a diagnosis of dementia in later life, suggesting that proactive management of modifiable conditions associated with the metabolic syndrome could reduce the risk of dementia [14].

## The Role of Advanced Practice in Global Health

APNs are frequently overlooked assets in the workforce despite their potential to improve healthcare delivery significantly. According to the International Council of Nurses (ICN), approximately 70 countries have either implemented advanced practice roles or are contemplating their introduction [15]. However, to credibly address global health, APNs need to actively acknowledge and work across boundaries to challenge the disparities in health outcomes between high‑income countries (HICs) and LMICs. The implicit acceptance of higher clinical risk and lower standards in areas with less political power must be rejected as unacceptable, and any efforts to impose processes that work in HICs must first be validated and tested in LMICs; otherwise, they risk being contextually irrelevant [16]. These disparities are a product of the social effects of colonialization, which was founded on violence, racism, misogyny, and Eurocentrism to establish a legacy of power and exploitation [17]. There is much debate in relation to the decolonization of global health and nursing, but this will not succeed without dismantling the underpinning global economy, which fuels socio‑economic inequity [18]. These asymmetries occur in relation not only to economies but also to disproportionately high rates of morbidity and mortality between HICs and LMICs and to the power structures that exist within the global health arena [19]. Abimbola and Pai [20] argue that all forms of supremacy in global health practice within countries, between countries, and all around the world must be removed for the decolonization of global health to be possible.

APNs in HICs must ensure that perspectives of Western cultural superiority are challenged and do not exclude the expertise and lived experiences of Indigenous and any other knowledge systems [17]. Within nursing education, beliefs and assumptions continue to be rooted in Western biomedical hegemony based on positivist beliefs despite the integration of holistic language [21]. This model perpetuates inequality and social injustice that global health seeks to dismantle. APNs need to be aware of the broader global structures and barriers in order to address global health. This is particularly important in relation to LMICs, where advanced practice is not as embedded into health systems, and APNs have the potential to make a significant impact here [41]. Academic institutions in HICs compete to generate income from delivering education in LMIC countries. Opportunities to contribute to the development of advanced practice in LMICs by APN academics must ensure that these programs are not constructed in line with colonial standards [16] and that learning is bidirectional and based on partnership [22]. APNs in HICs can utilize their leadership capabilities to progress work to break down barriers to the achievement of equitable health outcomes the world by having an understanding of the consequences of colonialism and for APNs across the globe to work together in a culturally safe environment [23], where power is equal, and imbalances in global health are redressed.

NPs make valuable contributions to global health. Advanced Practice Providers/NPs improve access to quality healthcare services, especially in underserved and rural areas with provider shortages, and provide culturally sensitive care to diverse populations globally as they learn to understand and adapt to the health practices, beliefs, and needs of different cultural and ethnic groups, promoting health equity in marginalized populations with barriers to accessing healthcare due to economic instability, rural areas, or other social determinants of health.

## Method

### Literature search

A comprehensive electronic literature search was conducted using PubMed, Medline/Ovid, and CINAHL databases. PubMed and Medline/Ovid are widely used to access a vast array of health‑related literature, and CINAHL focuses explicitly on nursing and allied health literature. A general Google search for websites and organizations was also conducted, and materials and articles were reviewed. Search terms include global health, advanced practice nursing, global health outcomes, and various combinations of the terms.

**Search outcome:** The search identified relevant studies in 118 peer‑reviewed articles published between 2018 and 2025. Evidence‑based guidelines recommend the use of the Preferred Reporting Items for Systematic Reviews and Meta‑Analysis (PRISMA) format [24]. The PRISMA flow chart provided in Appendix identifies the search outcomes and selection process in a visual format. The quality appraisal process involved assessing the methodological rigor and relevance of each study to ensure that only high‑quality research was included. Quality assessment of studies is a significant component of a robust review of evidence [25]. A quality assessment will provide a guide as to the strength and relevance of the reviewed research. This is important when considering the credibility and relevance of the study to practice [25].

**Data abstraction:** Abstracts were reviewed to seek advanced practice roles, impact, and outcomes on global health, as well as gaps in the current literature. Relevant information from each article was reviewed based on the findings of various studies and peer‑reviewed articles. (See Appendix 1: Prisma and Appendix 2: Evidence Table).

## Discussion

Research has shown that APNs/NPs have positive outcomes by providing high‑quality care, achieving high patient and family satisfaction, and spending more time with patients and families. APNs/NPs have also provided timely healthcare delivery and enhanced continuity of care [26–29]. Aiken et al. [27] study showed that NP employment in a large hospital demonstrated value‑added for inpatients. NPs had significantly better outcomes, including lower mortality, fewer readmissions, shorter lengths of stay, higher patient satisfaction, and favorable outcomes on other quality indicators [27]. The United States, Russia, and South Africa have also reported higher patient satisfaction rates with nurse‑led care compared to physician‑led care [30]. In India, where one‑quarter of approved physician positions remain vacant, addressing the provider shortage gap involves licensing APNs/NPs to deliver primary and preventive healthcare services under the supervision of medical doctors, thus enhancing healthcare [30].

In Oman, limited access to adequately prepared healthcare providers in primary healthcare was the driving force behind the implementation of the advanced practice nurse/NP role to address healthcare providers [31]. Ninety‑three percent of the population would travel abroad to receive quality healthcare [31]. The introduction of the APN/NP role received strong stakeholder support. NP care was notable for a holistic approach that emphasized health promotion, disease prevention, and compassionate, person‑centered care. This approach addressed all dimensions of health—social, physical, cultural, psychological, spiritual, and cognitive—leading to improved quality of care and greater satisfaction among patients and their families.

Four African countries have embraced the role of APNs/NP, and other countries, primarily in the sub‑Saharan region, have expressed considerable interest. The interest is driven by a shortage of medical practitioners and the inequitable distribution of the health workforce [32]. APN/NP can address the disparities in health provision many rural communities face. Regulation, education, and integration of the role are part of their struggle [33].

Like many other countries, Israel has limited resources, a shortage of qualified personnel, and an aging population. The Israeli Ministry of Health has set a target for NPs to comprise 6% of the national nursing workforce by 2030. As of 2022, significant progress has been made toward this goal, with NPs increasingly integrated into the healthcare system and playing a vital role in service delivery across the country. The Israeli Ministry of Health has consequently developed a unique specialization model for NPs in each clinical field (e.g., diabetic care, geriatric care, neonatal care, and pain control) [34].

APNs can contribute to global health initiatives in several significant ways:

They provide essential primary care services in underserved and resource‑limited regions, thereby mitigating the shortage of healthcare professionals and enhancing access to care. Their focus on preventive care and health promotion enables them to collaborate closely with local communities, implement culturally sensitive interventions, educate populations, and empower individuals to manage their health effectively. Playing a pivotal role in training and mentoring local healthcare workers, fostering sustainable healthcare systems, and elevating the overall quality of care delivered, they can contribute to global health research by i informing evidence‑based practices and policies tailored to the diverse healthcare needs of various populations.

APNs are invaluable assets in disaster relief and humanitarian efforts, delivering crucial healthcare services and aiding in the recovery and resilience of affected communities. Events like pandemics, natural disasters, and mass violence underscore the critical role of NPs in response and recovery. In a study by Morley et al. [35], APNs in England rapidly filled a clinical need caring for older people who were increasingly vulnerable to the effects of isolation and the delay of medical treatment during the COVID‑19 pandemic [35].

The advanced practice role is growing and is recognized as a viable alternative to addressing healthcare provider shortages, especially in low‑ and middle‑income areas. APNs/NPs, when appropriately educated, can provide emergency care to improve the quality of care and patient outcomes in countries such as Africa [36]. APNs can be cruciale in triaging and managing patients, coordinating care, and educating patients and families [29]. APNs in emergency care continue to evolve, and they are present in diverse emergency care settings in the USA, UK, Ireland, Australia, and many other countries [36]. Emergency preparedness education, conducting on‑site simulations, and providing training in adaptive management skills are essential to enabling APNs/NPs to lead effectively during crises.

## The Unique Contributions of Nurse Practitioners

A recently published umbrella review [2] found that APNs, NPs, and clinical nurse specialists provided care that was of equivalent or better quality when compared across several categories and a wide range of clinical settings, patient populations, and acuity levels, indicating that APNs are uniquely placed to influence global health concerns. APNs bring unique skills and expertise to the global health arena. Their advanced education and training equip them with the knowledge and competencies to provide high‑quality, comprehensive care across the lifespan. APNs are trained to diagnose and manage various acute and chronic conditions, prescribe medications, and order and interpret diagnostic tests [26, 37]. Moreover, APNs and NPs are well‑versed in health promotion, disease prevention, and patient education, which are essential to global health initiatives, as well as to leadership and challenging the status quo to reshape care, encompassing not only reactive condition management but also longer term preventative approaches. Their holistic approach to care, which considers the physical, mental, and social well‑being of individuals and communities, aligns with global health principles [3, 28].

**Examples**

- In Slovenia, efforts are underway to establish the APN role in family practices to help fill gaps in primary care access. A systematic review found that APNs contribute to improved patient outcomes in postoperative cardiac settings, although more research is needed [38].
- India has a population of approximately 1.3 billion people, and nurses are at the center of India’s healthcare system. Because the Indian healthcare system is overburdened by population, there is a lack of adequate workforce and an ineffective referral system. NPs have been emerging as a key resource in home‑based care referral and filling the gaps in primary care [30].
- In Norway, APNs were found to be as safe as physicians in diagnosing and treating patients with minor orthopedic surgeries in emergency care [3].
- In England, lung cancer patients assessed by APNs had a 17% lower risk of death and fewer unplanned cancer‑related admissions compared to those not assessed by APNs [3].
- In Ireland, APNs positively contributed to individual, family, and community outcomes for people with intellectual disabilities, a growing area of research and practice [3].
- APNs are expanding their roles in mental healthcare, delivering psychosocial health promotion, prevention, and consulting/coaching services [3].
- APNs have been successfully deployed in developed countries such as the USA, Canada, the UK, Australia and developing countries such as Kenya to improve access to primary and preventive healthcare services [37].
- APNs in the USA played a vital role in responding to the increased healthcare demands during the pandemic [39].
- Research demonstrates that APN‑delivered care is associated with positive patient outcomes across various specialties and settings, such as orthopedic surgery, oncology, mental health, and emergency care [2, 3].
- A systematic review found that APNs contribute to improved patient outcomes in postoperative cardiac settings, although more research is needed [3, 29].
- APNs have the potential to improve the health of underserved populations worldwide significantly [40].
- APNs can provide cost‑effective solutions to existing healthcare access and quality problems, particularly in areas with provider shortages [40].

While the APN role is at different stages of development and implementation across countries, these examples highlight their global contributions in expanding access, responding to emergencies, improving outcomes, promoting patient‑centered care, and addressing healthcare disparities through their advanced clinical expertise.

## Conclusion

Evidence consistently demonstrates the effectiveness of NPs in improving quality measures for patients across various healthcare settings [2]. As the global healthcare landscape continues to evolve, the demand for skilled and adaptable professionals like NPs is expected to rise. APNs in HICs have a moral obligation to achieve global health by working in true partnership to achieve health equity in LMICs. The global annual growth of the NP workforce has been estimated to be between three and nine times more significant compared to physicians [29]. Leveraging their expertise and commitment to holistic care, NPs can substantially enhance the health and well‑being of populations worldwide. By empowering APNs, healthcare systems can better address the diverse and evolving health needs of global populations, ultimately contributing to the achievement of universal health coverage and improved health equity worldwide.

## Additional Files

The additional files for this article can be found as follows:

Supplementary Appendix 1. PRISMA.

Supplementary Appendix 2. Evidence Table.
