# Supplementary figures and images for: Empowering Advanced Practice Nurses: A Review of Addressing Global Health Needs
Source: Ann Glob Health. 2025 Aug 13;91(1):45. doi: 10.5334/aogh.4723 (PMC12352385; doi:10.5334/aogh.4723)

Appendix 1 PRISMA

PRISMA

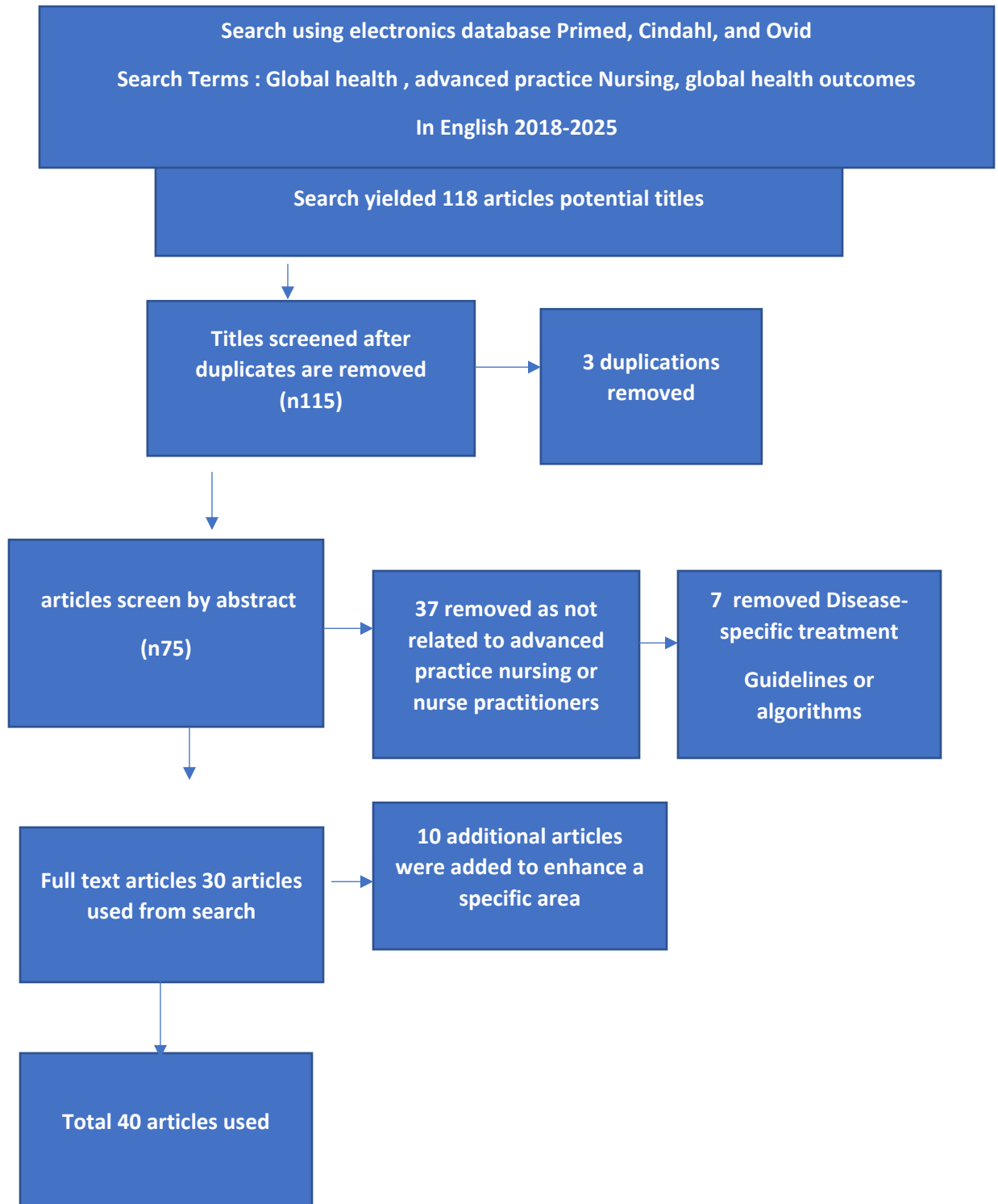

Supplement: Supplementary Appendix 1. — PRISMA. [file agh-91-1-4723-s1.pdf]
