# Supplementary material for: Empowering Advanced Practice Nurses: A Review of Addressing Global Health Needs
Source: Ann Glob Health. 2025 Aug 13;91(1):45. doi: 10.5334/aogh.4723 (PMC12352385; doi:10.5334/aogh.4723)
Supplement: Supplementary Appendix 2. — Evidence Table. [file agh-91-1-4723-s2.pdf]

Appendix 2 Evidence Table

**Authors/year/region(s)**	**Study design**	**Limitations**	**Aim**	**Methodology**	**Main findings**
Abimbola & Pai, 2020 Multinational	Not applicable (the paper is a theoretical discussion and does not involve a study design)	Small sample size in initial studies<br><br>- Limitations of Mendelian randomization in capturing short-term effects<br><br>- Need for further investigation into ketogenic diets as a weight-loss strategy in COVID-19 context<br><br>- Need for more research on variability in health response to diet during the pandemic	The aim is to decolonize global health by removing all forms of supremacy and achieving equity and justice within global health practices. Objectives include addressing the colonial origins of global health, transforming its structures, and ensuring that global health practices are inclusive, equitable, and just. The ultimate goal is a radical transformation of global health to meet its mission.	Not applicable (the paper is a conceptual discussion and does not employ a specific research methodology)	- Global health is in the early stages of a necessary decolonization process to remove supremacist structures and attitudes.<br><br>- The future of decolonized global health should be characterized by equity, justice, and the absence of supremacy.<br><br>- Superficial changes without addressing underlying supremacy will result in the failure of global health's mission.

Authors/year/region(s)	Study design	Limitations	Aim	Methodology	Main findings
Basu et al, 2017 Uganda, South Sudan, Liberia	Not mentioned (the paper is a commentary and does not describe a specific study design)	Not mentioned (the paper does not provide any explicit limitations or suggestions for further research)	The aim of the paper is to deconstruct specific learnings that have benefited the Johns Hopkins AI community through the APPS partnership, and to explore the role of South-North partnerships in promoting shared learning and knowledge transfer.	- Descriptive analysis of partnership experiences.<br><br>- Focus on bi-directional learning through the African Partnerships for Patient Safety (APPS) program.<br><br>- Involves interactions (in-person and virtual) between Johns Hopkins Medicine/Armstrong Institute and hospitals in Liberia, Uganda, and South Sudan.<br><br>- Deconstruction of specific learnings from these interactions.	- South-North partnerships in healthcare can lead to bi-directional learning, where high-resource hospitals benefit from innovations in low-resource settings.<br><br>- Lessons from African partners include people-centered care, waste reduction, and environmental consciousness, which have been adopted by high-resource hospitals.<br><br>- The concept of "reverse innovation" should be reframed as "global innovation flow" to emphasize mutual learning and shared benefits.

**Authors/year/region(s)**	**Study design**	**Limitations**	**Aim**	**Methodology**	**Main findings**
Clark et al., 2023 United Kingdom	Not mentioned (the paper describes a podcast and article series rather than a traditional scientific study with a specific design)	- Inability to control all variables in real-world clinical work.<br><br>- Lack of long-term RCTs in diet research due to expense and time consumption.<br><br>- No long-term evidence for current guideline-driven dietary strategies for diabetes management.<br><br>- Misunderstanding and undervaluation of clinical audits compared to RCTs.	The aim is to examine progress towards decolonialism in health and medicine, discuss the current state of the movement, and center the perspectives of those marginalized by colonialism. Objectives include amplifying multidisciplinary perspectives, highlighting the impact of colonial histories on healthcare, and advancing conversations on health justice and equity.	- Release of a podcast and article series to examine decolonialism.<br><br>- Centering perspectives and voices of people marginalized by colonialism.<br><br>- Amplifying multidisciplinary perspectives from scholars, practitioners, and advocates.<br><br>- Engaging with leaders from colonial era institutions.<br><br>- Using dialogue and discussion to explore the impact of colonial histories.<br><br>- Reflecting on positionality and privilege of the authors.	- Colonial legacies have deeply influenced modern health systems, resulting in flawed healthcare practices and structural inequities.<br><br>- Decolonization requires restoring power to marginalized groups and addressing historical inequities beyond superficial changes.<br><br>- Health professionals need education on colonial histories, and institutions must shift power to community-based expertise for meaningful change.

**Authors/year/region(s)**	**Study design**	**Limitations**	**Aim**	**Methodology**	**Main findings**
Kilpatrick et al., 2024 Multinational (38 countries, such as Africa, Europe, Southeast Asia, Eastern Mediterranean, Western Pacific, and Latin America)	systematic review of systematic reviews (overview), including randomized, non-randomized, and qualitative methodologies	- High levels of poverty amidst high fertility<br><br>- Disproportionately high burden of morbidity and mortality<br><br>- Unfavourable education outcomes<br><br>- High donor dependence<br><br>- Limited power in the global (health) arena<br><br>- Lack of introspection and self-awareness among African researchers<br><br>- Attempts to transplant best practices without considering contextual realities<br><br>- Treatment of Africa as a	Aim: To identify gaps in advanced practice nursing research globally. Objectives: 1. Identify the countries included in systematic reviews of APNs, NPs or CNSs; 2. Describe the types of included studies, study population, role definitions, and settings identified in the systematic reviews; 3. Examine the types of outcomes of APN, NP or CNS roles included in systematic reviews globally.	- Conducted a review of systematic reviews.<br><br>- Searched multiple databases (CINAHL, Embase, etc.) from January 2011 onwards.<br><br>- Included grey literature and hand searches.<br><br>- Used the Critical Appraisal Skills Program (CASP) for quality assessment.<br><br>- Study selection and data extraction were done independently by two reviewers.<br><br>- Developed a narrative synthesis using an iterative process.<br><br>- Followed a pre-published protocol registered with PROSPERO.	- Advanced practice nursing roles (APNs, NPs, CNSs) are generally equal or superior to traditional care models across 29 indicator categories.<br><br>- Mixed findings were noted in areas such as quality of life and healthcare service delivery.<br><br>- Emerging research areas include AI, with identified gaps in interprofessional team functioning and patient engagement.

Authors/year/region(s)	Study design	Limitations	Aim	Methodology	Main findings
		homogeneous entity			

Authors/year/region(s)	Study design	Limitations	Aim	Methodology	Main findings
Koplan et al., 2009 Multinational	Not mentioned (the paper is a viewpoint article focused on defining global health, not an empirical study with a specific design)	- Reflexivity: Researchers' experiences and assumptions might have influenced data.<br><br>- Altered communication: Participants' awareness of the study might have changed their behavior.<br><br>- Lack of participant feedback: Future studies should include participant comments on transcripts.<br><br>- No screening for expertise: Participants were not screened for expertise in person-centered care.<br><br>- Unconsidered variables: Did not	The aim of the paper is to define global health and advocate for the adoption of a common definition to ensure clarity and alignment in strategies and priorities among stakeholders.	- The paper is a viewpoint article.<br><br>- It presents reasoning and a definition agreed upon by a panel of multidisciplinary and international colleagues.<br><br>- The Consortium of Universities for Global Health (CUGH) Executive Board developed, reviewed, and edited the manuscript.<br><br>- All authors contributed to writing and editing the manuscript.	- Global health is defined as an area for study, research, and practice prioritizing health improvement and equity worldwide.<br><br>- It emphasizes transnational health issues and interdisciplinary collaboration and combines population-based prevention with individual clinical care.<br><br>- The paper calls for the adoption of a common definition to enhance global health efforts.

Authors/year/region(s)	Study design	Limitations	Aim	Methodology	Main findings
		account for distribution of evaluations, nurse experience, or gender.<br><br>- Contextual differences: Transferability requires consideration of contextual differences.			

**Authors/year/region(s)**	**Study design**	**Limitations**	**Aim**	**Methodology**	**Main findings**
Kwete et al., 2022 Wuhan, China; Asia; Latin America; Africa; multinational	Not mentioned (the paper is based on a symposium discussion and does not follow a specific study design)	Not mentioned (the paper does not explicitly list any limitations)	The aim of the paper is to explore the decolonizing global health movement by taking a post-colonial perspective on global health research and practice, with the goal of promoting equity and justice. It seeks to identify and categorize colonial remnants in global health at the levels of practices, institutions/organizations, and policies to guide systemic reforms for a fundamental paradigm shift.	- The methodology involved summarizing discussions from a symposium held in July 2021 in Wuhan, China.<br><br>- Experts discussed the meaning, methods, and criteria for decolonizing global health.<br><br>- The approach was qualitative, focusing on reaching a consensus and categorizing colonial remnants in global health into three levels: practices, institutional/organizational, and policy.	- The current global health system is influenced by colonial ideologies and practices, requiring systemic reforms for true decolonization.<br><br>- Three levels of colonial remnants in global health are identified: practices, institutional/organizational structures, and policy levels.<br><br>- Decolonizing global health is linked to broader socioeconomic changes, particularly equitable economic ownership of global wealth.

Authors/year/region(s)	Study design	Limitations	Aim	Methodology	Main findings
Lotta et al., 2015 Europe, East Asia	Systematic review, meta-analysis, observational study, prospective cohort study	- Lack of consensus on effective measures to address metabolic syndrome.<br><br>- Current prevention strategies may not adequately target younger populations.<br><br>- Need for comprehensive public health plans to combat metabolic syndrome.<br><br>- Limited accessibility and affordability of healthy lifestyle education and resources.	The aim of the study is to assess the predictive relevance of various definitions of metabolic health in relation to the risk of type 2 diabetes within different BMI categories. The primary objectives are to assess the risk of type 2 diabetes associated with current definitions of metabolic health within lean, overweight, and obese categories, and to evaluate the predictive relevance of these definitions.	- Systematic search of MEDLINE for prospective cohort studies.<br><br>- Two-stage meta-analysis: network meta-analysis followed by random-effects models.<br><br>- Use of hierarchical summary receiver operating characteristic curves for predictive performance assessment.<br><br>- Probabilistic analysis for cumulative incidence estimation.<br><br>- Statistical analyses conducted using STATA software.	- Individuals classified as metabolically unhealthy have a higher relative risk of type 2 diabetes across all BMI categories.<br><br>- Current binary definitions of metabolic health are not effective for predicting future type 2 diabetes.<br><br>- Metabolically healthy obese individuals still have a significant absolute risk of developing type 2 diabetes over 10 years.

Authors/year/region(s)	Study design	Limitations	Aim	Methodology	Main findings
Mcgibbon et al., 2014 Canada, South Africa, Jamaica	Not mentioned (the paper is a discussion article and does not describe a study design)	- Need for ongoing empirical data and policy change<br><br>- Additional empirical data needed for cost-effective integration<br><br>- Limited monitoring and effectiveness evaluation in some regions<br><br>- Need for more comprehensive data on NP effectiveness	The aim of the paper is to underscore the urgent need to further articulate postcolonial theory in nursing and to contribute to nursing knowledge about paths toward decolonizing the profession. It also aims to provide strategies to increase the counter-narrative against colonization, focusing on critical social justice, human rights, and structural determinants of health.	The paper uses a discussion-based methodology, focusing on theoretical and conceptual analysis of postcolonial theory as it applies to nursing. It is informed by previous research work in a federally funded project and involves critical analysis of existing literature and theories.	- The paper highlights the urgent need to integrate postcolonial theory into nursing to decolonize the profession.<br><br>- It identifies colonization processes within nursing, including intellectual development colonization and the role of white privilege and racism.<br><br>- Strategies for increasing counter-narratives focus on critical social justice, human rights, and addressing structural determinants of health.

Authors/year/region(s)	Study design	Limitations	Aim	Methodology	Main findings
Nashwan et al., 2024 Africa	Not mentioned (the paper does not specify a study design)	- Need for recognition and understanding of the role of APNs<br><br>- Limited resources and funding<br><br>- Shortage of trained and skilled APNs<br><br>- Lack of curricula in emergency care<br><br>- Limited trainers to run courses<br><br>- Resistance from healthcare organizations and policymakers<br><br>- Regulatory barriers<br><br>- Cultural and social barriers<br><br>- Lack of collaboration between healthcare providers<br><br>- Lack of training institutions	The aim of the paper is to highlight the vital importance of investing in advanced practice nursing (APN) for enhancing emergency care throughout Africa, explore the potential benefits of such investments, and promote further investment to advance African emergency care.	The paper uses a review methodology, drawing evidence from existing literature and successful implementations of APN in various healthcare environments to discuss the potential benefits and challenges of investing in APNs in emergency care in Africa.	- Investing in APNs in emergency care improves quality and access to care, leading to better patient outcomes.<br><br>- APNs contribute to reduced mortality rates and shorter hospital stays in emergency settings.<br><br>- Investment in APNs enhances the healthcare workforce by providing professional development opportunities.

**Authors/year/region(s)**	**Study design**	**Limitations Aim**	**Methodology**	**Main findings**
		- Need for further research to evaluate impact and identify best practices		

**Authors/year/region(s)**	**Study design**	**Limitations**	**Aim**	**Methodology**	**Main findings**
Oster & Chaves, 2023 United States, Asian countries	- Contribution 1: Case-control study<br><br>- Contribution 2: Longitudinal observational study<br><br>- Contribution 3: Observational cohort study<br><br>- Contribution 4: Observational study<br><br>- Contribution 5: Observational study<br><br>- Contribution 7: Experimental study (small-scale laboratory setup)	- Inconsistent study selection by two reviewers<br><br>- Language and geographical restrictions applied<br><br>- Low identification of all important studies<br><br>- Lack of clarity on second reviewer verification of extractions<br><br>- Challenges in identifying studies due to "nurse-led" terminology<br><br>- Lack of recognized APN, NP, or CNS titles in some countries<br><br>- Review quality downgraded due to methodological issues	The aim of the paper is to explore the effects of healthy lifestyle factors, such as diet, sleep, and exercise, on chronic diseases, with a particular focus on the role of timing and behavioral rhythms in enhancing health outcomes and preventing or managing metabolic disorders and cancer.	- Cai et al.: Case-control study with 410 Han Chinese individuals examining dietary patterns and gut microbiota enterotypes.<br><br>- Bianco et al.: Longitudinal study over one year on Mediterranean diet and exercise effects on glucose metabolism in MASLD patients.<br><br>- Gu et al.: Cohort study on 24-hour behavioral rhythms and MASLD in 4502 overweight/obese adults.<br><br>- Nitta et al.: Use of a food logging mobile app to compare meal patterns in Japanese participants over one month.<br><br>- Yang et al.: Investigation of sex-specific differences in diet and T2DM	- A healthy lifestyle, including diet, exercise, and sleep, is crucial for preventing and controlling chronic diseases.<br><br>- Lifestyle interventions are effective treatments for metabolic diseases such as MASLD.<br><br>- There are sex-specific effects of diet on the development of type 2 diabetes mellitus.

**Authors/year/region(s)**	**Study design**	**Limitations**	**Aim**	**Methodology**	**Main findings**
				risk using demographic and dietary intake data.<br><br>- Meyhöfer et al.: Small-scale laboratory study on sleep phase timing and its effects on appetite and hunger regulation.	

**Authors/year/region(s)**	**Study design**	**Limitations**	**Aim**	**Methodology**	**Main findings**
Rix et al., 2024 Australia	Not mentioned (the paper is a narrative account and does not specify a formal study design)	- Invasive biological sampling required for defining metabolic health.<br><br>- Limited value of additional criteria beyond fasting glucose.<br><br>- Limited predictive relevance of current binary definitions of metabolic health.<br><br>- Potential for more comprehensive approaches to improve predictive performance.<br><br>- Variability due to pooling evidence from studies with different definitions.	The aim of the paper is to explore the journey of white nurse educators in becoming genuine accomplices to Indigenous Peoples by promoting cultural safety and decolonization in healthcare. The paper seeks to address systemic resistance to cultural safety practices, emphasize the role of critical reflection, and highlight the importance of collaboration between Indigenous and white educators. It aims to support a lifelong process of unlearning for white nurse educators to reduce health inequity and increase cultural safety.	The methodology is primarily narrative and reflective, focusing on personal journeys and experiences of the authors as white nurses working alongside Indigenous Peoples. It involves collaboration with Indigenous co-authors and community members, using participatory approaches such as yarning to guide culturally safe practice and education.	- White nurse educators must become genuine accomplices, moving beyond performative allyship, to support Indigenous Peoples' struggle for health equity.<br><br>- A decolonizing approach in nurse education is fundamental to addressing systemic racism and promoting cultural safety.<br><br>- Effective teaching of cultural safety requires collaboration between Indigenous and white educators at the cultural interface.

Authors/year/region(s)	Study design	Limitations	Aim	Methodology	Main findings
Rosa et al., 2020 Multinational (North America, Europe, the Caribbean, Australia, New Zealand, Africa, Thailand)	Not mentioned (the paper does not specify a study design)	- Lack of an established definition of global health<br><br>- Potential confusion and misalignment in strategies and priorities among stakeholders<br><br>- Need for a common definition to ensure agreement on goals, approaches, skills, and resource use	To argue that nurse practitioners have been under-utilized generally in the current global health environment, creating barriers to achieving universal health coverage and the Sustainable Development Goals.	- Literature review using sources such as PubMed, Google Scholar, and reports from international organizations.<br><br>- Expert opinion based on the authors' experiences.<br><br>- No specific empirical study or data collection mentioned.	- Nurse practitioners are under-utilized globally, which creates barriers to achieving universal health coverage and the Sustainable Development Goals.<br><br>- Expanding the scope of practice for nurse practitioners can leverage their skills to improve healthcare access and quality.<br><br>- Ongoing empirical data and policy changes are needed to enable the full utilization of nurse practitioners in healthcare systems.

**Authors/year/region(s)**	**Study design**	**Limitations**	**Aim**	**Methodology**	**Main findings**
Saklayen, 2018 Bangladesh, USA	Not mentioned (the paper is a review article and does not describe a specific study design)	- Vastness and complexity of the decolonizing project<br><br>- Lack of integration of postcolonial ideas into nursing's consciousness or political agenda<br><br>- Reluctant and inconsistent embrace of structural determinants of health<br><br>- Positivist, individualistic, and Eurocentric foundations as barriers to progress	The aim of the paper is to highlight the global epidemic of metabolic syndrome and propose a societal, governmental, and global approach to control and prevent it through lifestyle changes and increased awareness.	The paper is a literature review and does not have a specific methodology section. It synthesizes information from various studies and data sources to discuss the prevalence, definitions, and contributing factors of metabolic syndrome.	- Metabolic syndrome is a global health issue, more prevalent in urban populations of developing countries than in Western countries.<br><br>- The primary drivers of metabolic syndrome are increased consumption of high-calorie, low-fiber foods and decreased physical activity.<br><br>- Approximately one quarter of the global population is estimated to be affected by metabolic syndrome, necessitating global efforts to change lifestyle habits.

Authors/year/region(s)	Study design	Limitations	Aim	Methodology	Main findings
Siouta et al., 2019 United Kingdom	Qualitative, observational, multi-site, non-participant observations, purposive sampling, discourse analysis, additional analysis of an existing ethnographic study	- Unclear interaction between dietary nutrient composition and gut microbiome composition in affecting colorectal cancer risk.<br><br>- Sex-specific effects of diet habits on T2DM development suggest limitations in generalizability.<br><br>- Need for further research to decipher molecular regulatory pathways of sleep and its physiological outputs.<br><br>- Small sample size in the study by Meyhöfer et al. (n = 15).	The aim of the study was to explore how person-centered and non-person-centered caring are verbally constructed in consultations between patients and nurses, specifically in nurse-led chemotherapy outpatient clinics.	- Qualitative study using audio-recorded observations.<br><br>- Discourse analysis to identify communicative patterns.<br><br>- Additional analysis of existing ethnographic data.<br><br>- Non-participant observations in nurse-led chemotherapy clinics.<br><br>- Use of an observation guide for non-verbal data collection.<br><br>- Semi-structured interviews with nurses for validation and insights.<br><br>- Analysis involved sorting data into themes using a discourse-analytical perspective.<br><br>- Purposive sampling of	- The dominant discourse in nurse-led consultations was non-person-centered, focusing on biomedical aspects.<br><br>- Fragments of person-centered discourse were identified, showing potential for more holistic care.<br><br>- Nurses often focused too much on biological markers, missing opportunities for person-centered care.

**Authors/year/region(s)**	**Study design**	**Limitations Aim**	**Methodology**	**Main findings**
			participants with relevant experience.	

Authors/year/region(s)	Study design	Limitations	Aim	Methodology	Main findings
Ssennyonjo et al., 2023 Africa	Not mentioned (the paper does not specify any study design characteristics)	- Need for more theoretical discussion to guide practices<br><br>- Lack of a systemic theoretical approach to post-colonial issues in global health<br><br>- Preliminary nature of the current framework, requiring further research to identify and specify problems and develop practical solutions	The aim of the paper is to explore how the decolonization of global health can be aligned with Africa's strategic aspirations, such as those outlined in Agenda 2063 and the Sustainable Development Goals, by addressing development challenges and fostering equitable partnerships between Africa and the Global North.	Not mentioned (the paper does not describe a specific methodology as it is more of a conceptual discussion)	- Africa's development requires decolonization efforts that address historical legacies and promote local solutions.<br><br>- Inclusive partnership models and collaboration with the global north are essential for capacity building in Africa.<br><br>- Investment in human capital, especially for Africa's youth, is crucial for achieving sustainable growth and development.

Authors/year/region(s)	Study design	Limitations	Aim	Methodology	Main findings
Stefan et al., 2021 Multinational (USA, Saudi Arabia, Turkey, Egypt, Libya, Iran, Iraq, South Africa, Canada, Mexico, Australia, South America, Europe, France, China, Germany)	Review article, including findings from observational studies, Mendelian randomization analysis, and a meta-analysis	- Positionality and privilege as mostly white editors from coloniser or settler countries<br><br>- Lack of expertise in decolonising health	The aim of the paper is to summarize data on the independent relationships of obesity, visceral obesity, and impaired metabolic health with severe COVID-19, discuss the consequences of SARS-CoV-2 infection on organ function and future cardiometabolic disease risk, and propose strategies to prevent and treat obesity and impaired metabolic health during the COVID-19 pandemic.	The methodology involves summarizing data from large observational studies, using Mendelian randomization analysis to explore causal relationships, and employing genome-wide association study (GWAS) data to support findings. The authors also use multivariate adjustment to analyze relationships between comorbidities and COVID-19 outcomes.	- Obesity, particularly severe obesity, is a strong and independent determinant of severe COVID-19.<br><br>- Hyperglycemia, even in non-diabetic ranges, strongly predicts severe COVID-19.<br><br>- Treating obesity and cardiometabolic complications may effectively reduce the risk of severe COVID-19 and post-COVID-19 cardiometabolic diseases.

**Authors/year/region(s)**	**Study design**	**Limitations**	**Aim**	**Methodology**	**Main findings**
Unwin, 2024 United Kingdom	Observational study (clinical audit)	Not mentioned (the paper does not provide any explicit limitations or suggestions for further research)	The aim of David Unwin's paper is to evaluate the effectiveness of current interventions for reducing overweight and obesity, explore alternative dietary strategies such as low carbohydrate diets, and critique the reliance on RCTs in nutrition science while promoting practice-based evidence through clinical audits.	- Initially provided standard dietary advice ('eat less, move more', low-fat diet).<br><br>- Shifted to recommending a low carbohydrate diet for patients with T2 diabetes and pre-diabetes.<br><br>- Conducted clinical audits to evaluate the effectiveness of dietary interventions.<br><br>- Emphasized the use of real-world data from audits rather than RCTs.	- A low carbohydrate diet resulted in a mean weight loss of over 10% body weight at 3 years in a clinical practice setting.<br><br>- In a related study, 25% of participants lost more than 10% of their body weight within a year using a low carbohydrate approach.<br><br>- Clinical audits are valuable for evaluating real-world interventions and should be considered alongside RCTs.

Authors/year/region(s)	Study design	Limitations	Aim	Methodology	Main findings
Yanful et at., 2023	Not mentioned (the paper does not specify a study design)	- The process of decolonising global health is only just beginning.<br><br>- Lack of a clear vision or consensus on goals and methods for decolonisation.<br><br>- Current efforts may be hindered by inadequate responses and lack of direction.<br><br>- The ideas presented are preliminary and not yet fully actionable.	The aim of the paper is to deliver high-quality healthcare for all by recognizing and addressing the legacies of colonialism that drive power asymmetries and produce inequitable health outcomes. The objectives include challenging lower standards of care in less powerful populations, highlighting double standards to push for systemic changes, and adopting a systems-led decolonial approach that responds to local needs and addresses systemic imbalances of power.	The paper does not employ a traditional research methodology. It is a conceptual article based on discussions from the Thematic Working Group on Health System Quality and is part of a commissioned collection by The BMJ. It focuses on theoretical perspectives and expert opinions rather than empirical data collection and analysis.	- Recognizing the legacies of colonialism is essential for addressing power asymmetries and inequitable health outcomes in healthcare systems.<br><br>- A decolonial approach requires systemic action and reflexivity to challenge lower standards of care for politically marginalized populations.<br><br>- Decolonizing healthcare education and culturally relevant quality measurement are critical steps towards achieving equitable healthcare.
